# Health Week at Middleton

**Published:** 1924-11

**Authors:** 


### HEALTH WEEK AT MIDDLETON.

Middleton's Health Week, organised by the combined efforts of Mr. Victor C. Wilde, Chairman of the Health Committee, and Major S. T. Beggs, Medical Officer of Health, appears to have been the model of what such a week should be. The centre of the Committee's activities was the exhibition held in the Co-operative Hall—an exhibition which should be of great practical help. There were shown, for instance, meals as they ought to be—breakfast of porridge and eggs, mid-day dinner of meat and potatoes, and so on through the various repasts of the day. There were exhibits of footgear and clothing suitable for children, gas stoves and boilers, an electric heater, a large meat safe made by a boy of fifteen. Round the walls were plans from the Middleton Surveyor's Department—foremost among them those of the proposed new baths—and posters pointing out the evils of drink. Then there was a chamber of horrors, a room containing photographs of meat in various stages of disease. Not least calculated to stir the people of Middleton into activity and care was the booklet containing such telling admonitions as, "If half as much was spent at the birth as at the funeral, fewer babies would die," "Don't turn out the room without first turning out the baby." The booklet appeals for greater interest in public health, increased cleanliness, more co-operation between school medical staffs and parents. It explains the aims of the health services, showing what has been accomplished, and points out the causes of infection and how to prevent it. Much stress is laid on the evil of dental decay and the importance of a knowledge of food values.

